# Erythromycin lacks colon prokinetic effect in children with functional gastrointestinal disorders: a retrospective study

**DOI:** 10.1186/1471-230X-8-38

**Published:** 2008-08-21

**Authors:** Narayanan Venkatasubramani, Colin D Rudolph, Manu R Sood

**Affiliations:** 1 Division of Pediatric Gastroenterology and Nutrition, The Children's Hospital of Wisconsin and The Medical College of Wisconsin, Milwaukee, WI 53226, USA

## Abstract

**Background:**

Motilin, a peptide hormone has a direct excitatory effect on circular smooth muscle strips derived from the human colon. Reduced plasma motilin concentration has been reported in adults with chronic constipation. Erythromycin, a non-peptide motilin receptor agonist, induces phase 3 of the migrating motor complex (MMC) in the antro-duodenum and also reduces oro-cecal transit time. A pediatric study has reported an improvement in clinical symptoms of constipation following erythromycin administration, but the effect on colon motility in children has not been formally evaluated. We used colon manometry to study the effect of intravenous erythromycin lactobionate at 1 mg/kg on colon motiltiy in ten children.

**Methods:**

We selected patients with normal antroduodenal and colon manometry studies that were performed simultaneously. All studies were performed for clinically indicated reasons. We quantified the effect of erythromycin on colon contraction by calculating the area under the curve (AUC).

**Results:**

The mean (SE of mean) AUC in the colon during the fasting, post-erythromycin and postprandial phases of the study was 2.1 mmHg/sec (0.35), 0.99 mmHg/sec (0.17) and 3.05 mmHg/sec (0.70) respectively. The AUC following erythromycin was significantly less compared to the fasting phase of the study (p < 0.01).

**Conclusion:**

Erythromycin lacks colon prokinetic effect in children with chronic constipation evaluated by colon manometry.

## Background

Constipation accounts for 3% of general pediatric and 10–20% of pediatric gastroenterology outpatient visits [1]. A majority of these patients have functional constipation and the symptoms improve following behavioral modification and laxative treatment [2]. Almost 30% of children with chronic constipation will have persistent symptoms or relapses that can persist into adult life [3]. Children with chronic intractable constipation, who do not respond to conventional medical therapy, require manometric evaluation to exclude an underlying colon neuromuscular abnormality.

Motilin is a 22-amino acid peptide hormone secreted by the enterochromaffin cells of the small intestine [4,5]. It exerts profound effect on gastric and small bowel motility by inducing the inter-digestive phase 3 of the migrating motor complex (MMC) [6]. Peak plasma concentration of motilin is associated with MMC, both in animals and humans [7-9].

Conflicting results exists regarding the erythromycin effect on circular smooth muscle strips derived from the human colon. Few studies have reported the stimulatory effect of erythromycin in human smooth muscle contractions [10,11] and Nissan have reported lack of any excitatory effect on human colon [12]. Studies have shown that motilin receptor is expressed in the enteric neurons of the colon [10,13] and plasma motilin concentration is reduced in adults with chronic constipation [14]. Oral and intravenous erythromycin has no effect on distal colon contraction or transit in healthy human volunteers [15].

Erythromycin is a non-peptide motilin receptor agonist which induces phase 3 of the migrating motor complex in the antro-duodenum. Several studies have reported that erythromycin is safe and effective in improving feeding intolerance in preterm infants and children [16-19]. The prokinetic effect of erythromycin has also been reported in older children with motility disorders [20]. The data regarding the colon prokinetic effect of erythromycin is controversial. Oral erythromycin has been shown to reduce the colonic transit time assessed using radio opaque markers in adults with chronic constipation [21]. However, another adult study using colon manometry reported no significant improvement in colon motility with erythromycin compared to a placebo [22]. To date, no studies have evaluated the effect of erythromycin on colon motility in children.

The aim of our study was to evaluate the effect of intravenous erythromycin lactobionate (1 mg/kg) on colon motility in children with chronic constipation and fecal incontinence using colon manometry.

## Methods

We retrospectively evaluated 10 simultaneously performed antro-duodenal manometry (ADM) and colon manometry (CM) studies performed at the Children's Hospital of Wisconsin between June 2000 and June 2005. These studies were performed to exclude an underlying small bowel and/or colon motility disorder. Only patients with normal antro-duodenal and colon motility studies were included. The presenting symptoms were chronic constipation in 8 patients, abdominal pain and fecal incontinence in 1 and abdominal distension and pain in 1 patient. Hirschsprung's disease was excluded either by anorectal manometry or rectal biopsies in all subjects. This retrospective chart review study was approved by the Children's Hospital of Wisconsin Human Research Review Board (Protocol number: CHW 05/187, GC 31). Informed consent was not obtained from the patients as this was a retrospective chart review study.

**Figure 1:**
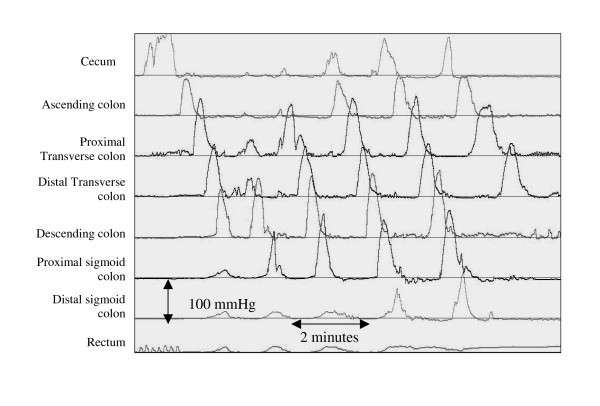
**HAPCs in a patient with chronic constipation**. High amplitude propagating contractions (HAPCs) following bisacodyl stimulation in a 10 year old boy with chronic constipation. The colon contractions are propagating from the cecum to the sigmoid colon.

All drugs known to affect the gastrointestinal motility were discontinued at least 72 hours before the motility studies. Patients fasted for at least 8 hours before the study. All children were anesthetized without a muscle relaxant. We waited for the child to recover completely from the effects of the drug before starting the motility tests [23]. Colonoscopy was performed to assist colon manometry catheter placement. The tip of the colon motility catheter was positioned in the cecum/ascending colon in all subjects and the position was confirmed by fluoroscopy. A water perfused manometry catheter with 8 recording sites was used for ADM and CM. Catheter position was checked using fluoroscopy. The catheter was perfused with 0.45% sodium chloride solution at the rate of 0.4 ml per minute per recording site, using a pneumo-hydraulic infusion system. The pressures were transmitted to a transducer and recorded on a computer with specialized motility software (Medical Measurements System, Amsterdam). We performed at least 2 hours fasting recording of both ADM and CM studies, following which we administered intravenous erythromycin lactobionate 1 mg/kg and performed another 60 minute of recording. Next, the patients ate a meal appropriate for their age (meal provided >30% of daily caloric requirement) and the recording was continued for another 60 minutes [24]. Bisacodyl (5–10 mg) was administered through the central lumen of the manometry catheter, directly into the colon to induce high amplitude colon contractions (HAPCs) and recording was continued for at least 30 minutes (Figure 1). This is a standard protocol for simultaneously performed ADM and CM studies performed at our center.

Antro-duodenal manometry was considered normal if i) phase 3 of the MMC, as defined by repetitive antral contractions occurring at a rate of 2–3 per minute and 10–12 per minute small bowel contractions, lasted for more than 2 minutes with normal antegrade propagation ii) there was normal stomach antrum and small bowel response to a meal [25]. Colon manometry studies were considered normal if there was a gastro-colonic response and spontaneous or bisacodyl stimulated antegrade propagating HAPCs [26]. We defined HAPCs as colon contractions with amplitude of at least 60 mmHg, and propagating over at least 30 cm of colon.

All colon manometry studies were reviewed by an experienced gastroenterologist and artifact was removed. The area under the curve was calculated by measuring the area under the pressure line for one 60 minutes period during fasting, following erythromycin administration and after a meal. Increase in motility index following a meal is considered a gastro-colonic response. The CM recordings were also evaluated for HAPCs.

We used SPSS software, version 10 for Windows (SPSS Inc., Chicago, Illinois, USA). We compared the mean (SE of mean) AUC using Student's t test. To compare the effect of erythromycin and bisacodyl on colon contractions, we used McNemar's test for disagreement.

## Results

The mean age of the patients at the time of the study was 9.6 years (range 4–12 years); there were five females. In all patients, we recorded phase 3 of the MMC following intravenous erythromycin and a normal postprandial antro-duodenal manometry.

The mean (SE of mean) AUC in the colon during the fasting, post erythromycin and postprandial phases of the study was 2.1 mmHg/sec (0.35), 0.99 mmHg/sec (0.17) and 3.05 mmHg/sec (0.70), respectively (Table 1). The AUC following erythromycin was significantly less compared to the fasting phase of the study (p < 0.01), suggesting that colon motor activity was reduced following erythromycin administration.

**Table 1:** AUC and HAPCs during fasting, following a meal and erythromycin

	Fasting Period	Postprandial period	Post-erythromycin period	Bisacodyl stimulation
Mean area under the curve in mm/sec (SE of mean)	2.1(0.35)	3.05 (0.70)	0.9 (0.17)	NA
Mean number of HAPCs (range)	0.4 (0–1)	0.00 (0)	0.1 (0–1)	8.9 (1–16)

The mean area under the pressure line during fasting, following a meal and erythromycin lactobionate administration. The mean number of HAPCs recorded during each of these periods and following bisacodyl stimulation are also shown

All patients had a normal gastrocolonic response. Four patients had spontaneous HAPCs during the fasting period, one following erythromycin stimulation and none following the meal. All patients had HAPCs with bisacodyl stimulation, the mean number of HAPCs was 8.9 (range 1–16). McNemar's test for disagreement between erythromycin and bisacodyl induced HAPC was statistically significant (p = 0.004). The mean interval of first HAPC after bisacodyl was 7.18 minutes (range 1–10 minutes).

## Discussion

This retrospective study evaluates the effect of erythromycin on colon motility in children with chronic constipation using colon manometry. All our patients had normal antro-duodenal and colon manometric studies. The two recognizable features of normal colon motility in children are an increase in colon contractions following a meal (gastrocolonic response) and HAPCs[26]. If spontaneous HAPCs are not recorded during fasting and postprandial period, bisacodyl is used to stimulate HAPCs [27]. All patients in our study had a normal gastrocolonic response and bisacodyl induced HAPCs. This suggests that none of our patients had intestinal pseudo-obstruction or colon neuromuscular abnormality. In our opinion, these patients had functional constipation and/or fecal incontinence.

All patients showed normal phase 3 MMC activity following intravenous erythromycin lactobionate (1 mg/kg dose). This shows that the dose of erythromycin lactobionate used was adequate to stimulate the motilin receptors in the foregut. In the colon there was a significant decrease in the frequency and amplitude of contractions following erythromycin lactobionate when compared to the fasting period. This may be because the motilin receptors in the colon have a higher threshold of activation compared to small bowel. An adult study reported no prokinetic effect of erythromycin on colon motility as determined by colon manometry [22]. This suggests that unlike the foregut, erythromycin lactobionate in a dose of 1 mg/kg does not have a prokinetic effect on the colon and probably a higher concentration may be necessary to stimulate the motilin receptors in the colon.

There is heterogeneity in the motilin receptor affinity for erythromycin in the gastrointestinal tract [28]. It is possible that the colon motilin receptors may have reduced affinity for erythromycin compared to the antral nerves or the expression of motilin receptor may be reduced in the colon. The limitation of our study is that we only evaluated the effect of a single dose of erythromycin (1 mg/kg) given intravenously. It is possible that a higher dose may be necessary to induce a prokinetic effect on the colon. An adult study using a higher oral dose of erythromycin (1 g/day), reported improvement in segmental and colon transit time assessed using radio-opaque markers [21].

## Conclusion

Our study suggests that erythromycin lactobionate at 1 mg/kg does not have a colon prokinetic effect in children with chronic intractable constipation. Further studies are needed, using a higher dose of erythromycin, to evaluate the dose response curve and affinity of the colon motilin receptors to erythromycin.

## Abbreviations

MMC: migrating motor complex, AUC: area under the curve, ADM: antroduodenal manometry, CM: colon manometry, HAPCs: high amplitude colon contractions

## Competing interests

The authors declare that they have no competing interests.

## Authors' contributions

All authors read and approved the final manuscript.

NVS: Data analysis and manuscript writing. CR: Helped with manuscript writing and providing patients. MS: Helped with manuscript writing, data analysis and providing patients.

## Pre-publication history

The pre-publication history for this paper can be accessed here:


